# The Perfect Family: Decision Making in Biparental Care

**DOI:** 10.1371/journal.pone.0007345

**Published:** 2009-10-13

**Authors:** Erol Akçay, Joan Roughgarden

**Affiliations:** 1 Department of Biology, Stanford University, Stanford, California, United States of America; 2 Current address: National Institute for Mathematical and Biological Synthesis (NIMBioS), University of Tennessee, Knoxville, Tennessee, United States of America; 3 Department of Biology, Stanford University, Stanford, California, United States of America; University of Utah, United States of America

## Abstract

**Background:**

Previous theoretical work on parental decisions in biparental care has emphasized the role of the conflict between evolutionary interests of parents in these decisions. A prominent prediction from this work is that parents should compensate for decreases in each other's effort, but only partially so. However, experimental tests that manipulate parents and measure their responses fail to confirm this prediction. At the same time, the process of parental decision making has remained unexplored theoretically. We develop a model to address the discrepancy between experiments and the theoretical prediction, and explore how assuming different decision making processes changes the prediction from the theory.

**Model Description:**

We assume that parents make decisions in behavioral time. They have a fixed time budget, and allocate it between two parental tasks: provisioning the offspring and defending the nest. The proximate determinant of the allocation decisions are parents' behavioral objectives. We assume both parents aim to maximize the offspring production from the nest. Experimental manipulations change the shape of the nest production function. We consider two different scenarios for how parents make decisions: one where parents communicate with each other and act together (the perfect family), and one where they do not communicate, and act independently (the almost perfect family).

**Conclusions/Significance:**

The perfect family model is able to generate all the types of responses seen in experimental studies. The kind of response predicted depends on the nest production function, i.e. how parents' allocations affect offspring production, and the type of experimental manipulation. In particular, we find that complementarity of parents' allocations promotes matching responses. In contrast, the relative responses do not depend on the type of manipulation in the almost perfect family model. These results highlight the importance of the interaction between nest production function and how parents make decisions, factors that have largely been overlooked in previous models.

## Introduction

The study of parental care is one of the most prominent areas in behavioral ecology. A large number of empirical and theoretical studies have been published attempting to explain patterns of parental care in terms of the fitness costs and benefits to the individuals supplying it [1]. These fitness costs and benefits are usually studied in the framework of sexual conflict, which posits that behavioral interactions between breeding males and females are driven by the conflicts between their evolutionary interests. Currently, the common wisdom seems to be that “the evolution of parental care is riddled with sexual conflict and a resulting evolutionary tug-of-war between males and females” [2, p. 156]. The most prominent prediction from this line of work is called partial compensation, and means that when parents respond to loss of effort by their partners, they should only do so partially. However, this prediction has had only mixed success in empirical tests. At the same time, theoretical work on the issue of how caring parents make their decisions in a social context remains scarce. In this paper, we aim to address these two issues. Our approach is to analyze the behavioral decisions made by parents under two different models of how they make decisions. We show that these two models yield a variety of predictions as a function of the ecology of nest production, which can explain the observed variation in experimental tests of the partial compensation prediction. Moreover, the two models also yield strikingly different response patterns to different model experiments, which allow empirically distinguishing between them. Below, we start by an overview of previous theory and its central prediction.

### Overview of previous theory

The central concept in parental care theory is parental investment (PI). Trivers [3] defined PI as any investment in an offspring that benefits that particular offspring, but prevents the parent from investing in other offspring. This definition is logically self-consistent as it defines PI in the relevant currency, namely offspring produced. However, it also makes PI a complicated variable that depends on population-level patterns of care and demography because these determine the opportunities to invest in other offspring [4]. Consequently, PI in Trivers' sense is hard to measure, and most empirical and theoretical studies focused on a different variable, parental effort (PE), defined as the material resources (such as time or energy) invested in or risks taken during parental care [5], [6].

A major question in parental care theory is what amount of PE should be invested by each parent in a biparental species (e.g. most birds). Since the 1970's, several models have been built to answer this question [5], [7]–[11]. In all these models, parents face a trade-off between investing time and energy into their current broods versus investing into their own survival or remating effort in order to gain other reproductive opportunities. These alternative reproductive opportunities are the cause of the conflict between parents' fitness interests. (In the special case of complete genetic monogamy for the lifetime of both parents, this conflict disappears, but this is generally regarded as a rare and derived case.) The fitness functions that result from this trade-off are then analyzed to find the stable levels of PE. There are different flavors to the different models: the one by Chase [8] is purely behavioral, and assumes that individuals reach optimal PE by reacting to each others PE behaviorally. Later, the analysis of Houston and Davies [9] modifies this assumption and allows the interpretation that individuals' PE are genetically determined. Finally, McNamara et al. [10] stipulate that individuals react to each other in behavioral time according to some genetically determined response rules, and attempt to calculate the evolutionary stable response rules. More recently, this model has been extended by Johnstone and Hinde [11] to include the possibility of imperfect information.

Despite the differences in how they treat the decision making by the parents, all of these models (except [11]) yield a common prediction. If parents are allowed to respond to each other behaviorally (as opposed to their efforts being genetically fixed), they should do so in a particular manner. Namely, if one of the parents decreases its effort, the other parent should increase effort in return, but by a smaller amount then the original decrease. This type of response is called *partial compensation*. To see the intuition behind this prediction, consider what would happen if the responding parent compensated fully for the loss of effort by its partner: the brood would then continue receiving the same level of total care. This means that the parent decreasing its effort does not experience a fitness reduction in the current reproductive success, while it benefits from the increased survival (or remating) chances. Consequently, that parent will enjoy higher fitness and will be selected to reduce its effort. The exception to this prediction is the model by Johnstone and Hinde [11], who predict response rules with positive slopes, i.e. a matching response, under some parameter regimes. The reason behind their prediction is that in their model, parents operate under uncertainty about the brood's real need. Therefore, changes in partner effort also convey information about changes in the brood's need, which under some conditions elicits a matching response. The relation between Johnstone and Hinde's model and the current one is taken up in more detail in the Discussion.

### Empirical tests

A number of empirical studies in birds have tested the prediction of partial compensation, this literature has been reviewed recently by Hinde [12]. The most common methods involve handicapping one of the parents by attaching small weights to their tails [13] or clipping some tail feathers [14]. The handicapped parent commonly (but not always, e.g. [15]) decreases the frequency of the food deliveries it makes to the nest, which is taken to be a measure of PE. The partner is then predicted to compensate partially by increasing its food delivery rate, but not as much as the original reduction by the handicapped parent. However, the results of these experiments show considerable variation, including no compensation (house sparrows, [16]), partial compensation (orange-tufted sunbird, [17]) and full compensation (great tits, [15]). Of the 10 handicapping studies that Hinde reviews 5 show no compensation, 2 partial compensation, and 3 full compensation. In the same article, Hinde reports on her own study that instead of handicapping one parent, simulated begging calls by nestlings in great tits. In this case, parents show a matching response, meaning that both of them adjust their provisioning rates in the same direction [12]. The contrast between the prominent partial compensation prediction from previous theory and the observed variation in empirical results motivates our model.

### A new approach

In this article, we develop a different approach to the question of how parents should respond to manipulations. There are two major sets of assumptions we make in our model. The first set concerns the ecology of parental care and how it affects the production of offspring from the nest. We assume that the nest production is dependent not only on the total PE, but also on the allocation of that effort between two competing parental tasks, which we take to be nest defense (or vigilance) and food delivery. We measure PE as the total time spent on care. Thus, parents need to allocate their time optimally between two tasks. Almost all previous manipulation studies have measured only one parental care component, provisioning, and equated changes in provisioning to changes in total PE. The exception to this trend is the study by Markman et al. [17], which measures both time spent provisioning and time spent on nest guarding, and documents a trade-off between these two. Here, we model a situation where changes in provisioning in response to manipulations can be explained by changes in allocation of PE.

We also incorporate explicitly the nature of the experimental manipulation into our model when predicting parents' responses. The nature of the manipulations turns out to be decisive in determining how parents respond to manipulations, but has largely been overlooked in previous models. There is again, one exception to this trend, which is the model by Sanz et al. [15], which explicitly incorporates two types of possible manipulations into their predictive framework.

The second set of assumptions concerns how parents make decisions. Our model operates at the behavioral, as opposed to evolutionary, time-scale. That is, we model parents that adjust their parental care decisions dynamically in response to changes in the environment, similar to Chase [8]. Parents' decisions affect how much the chicks grow and how likely they are to survive, and how much energy reserves the parent has left. These factors result in a fitness accumulation rate from the nest for each parent, which can then be incorporated in a demographic equation for natural selection. We then assume that parents have objective functions that they aim to maximize by behaviorally adjusting their allocations. These objective functions are proximate causes of allocation decisions, distinct from evolutionary fitness, which represented by the fitness accumulation rate. The notion of behavioral goals and objectives, as distinct from an individual's fitness, has been an integral part of ethology for a long time (e.g. [18]) but has lost its prominence in modern behavioral ecology. Our analysis re-emphasizes behavioral objectives as proximate causes for individuals' actions. We conjecture that it is these behavioral objectives that are evolved traits of individuals, rather than any specific PE or allocation decision. This follows the argument by Roughgarden et al. [19], who proposed that behavioral decisions should be modeled at the behavioral time-scale, which in turn is to be embedded in an overarching evolutionary genetics tier.

Recently, Akçay et al. [20] have developed a general framework for finding evolutionarily stable behavioral objectives. Shortly, their framework considers an interaction where each individual acts as to maximize a certain objective (for example, its own payoff). These objectives lead to a within-generation behavioral dynamics that then arrive at a certain set of actions at the equilibrium, where individuals locally maximize their own objectives. The payoffs from the behavioral equilibrium are then taken as the fitness of the individuals. Then, a mutant with a different objective function than the resident is introduced, and its fitness is calculated using the new behavioral dynamics that ensue. In this manner, they derive equations for behavioral objectives that are evolutionarily stable, i.e. cannot be invaded by any mutant.

In this paper, we skip this evolutionary analysis, and instead aim to produce a predictive framework for parental manipulation experiments, which exclusively take place in the behavioral time scale. For that, we need to specify what the evolved objectives of parents might be. Here, we assume that both parents aim to maximize the number of offspring fledged from the nest, which we call the nest production. This choice means that parents objectives are concordant, at least at the time-scale of manipulations. There are three independent motivations for this choice. The first motivation is intuitive: nest production is a simple and measurable measure of parental success, and is undoubtedly a major, if not the sole determinant of parental decisions at the behavioral scale we consider in our model. The second motivation comes from the results of Akçay et al. [20], who show that in games where players have conflicting payoff interests, efficient outcomes (i.e. ones upon which it is not possible to improve both players' payoffs) can be achieved only if parents' objectives are locally concordant at the behavioral equilibrium. In finite populations, only efficient outcomes are long-term evolutionarily stable [21], [22], which implies that evolution can frequently lead to objectives that are concordant (see Discussion for more on this). Finally, the third motivation for this choice is methodological; we are interested in asking what predictions parents having concordant objectives leads to, and whether this case can account for the observed in empirical studies. In general, the objective functions can also engender some conflict between parents, and the case we consider can be viewed as a null-model to compare the conflict model with.

A second question we are interested in is whether parents act jointly, meaning that they communicate about factors affecting their decisions and decide on their allocations together, or whether they act independently from each other. This question is different than whether parents have common objectives. Even in the case where both parents aim to maximize offspring production from the nest, they might still face uncertainty about what the optimal allocation is. Parents might have different information about factors such as the brood's hunger level, temperature, or predation pressure, which will affect what their perception of the optimal time allocation. This is an important consideration for manipulation experiments: if one of the parents is manipulated while the other is not, the manipulated parent will experience a different environment than the non-manipulated parent. We model two ways in which the parents might arrive at their allocations. One is where parents communicate with each other, agree on a common picture of the environment, and act accordingly. In this case, they will maximize the same nest production function, which we label “the perfect family,” corresponding to the most harmonious state of affairs between parents. The second way is one where each parent decides its allocation individually, according to its own information about the environment. This we call the “almost perfect family:” even though parents have the same objectives, they do not actually work together to achieve it.

In the next section we describe our model and the analysis method we use for the two different scenarios of how parents decide on their allocations. We then present the conditions under which parents should compensate for changes in each other's allocation in response to manipulations under the two scenarios. We end with a discussion of the assumptions of our model as well as how to distinguish between the two different decision making scenarios in our model.

## Analysis

Imagine two parent birds at a nest that must allocate time between two competing parental activities, say, nest defense and offspring provisioning. Parents adjust their allocations dynamically in a short time frame (e.g. minutes or hours) to maximize offspring production from the nest. When deciding on their allocations, parents face a budget constraint: they each have a fixed time allocated for parental activities, which we denote by 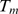 and 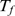 for the male and the female, respectively. The time budgets 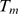 and 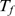 are in effect measures of total PE. We regard the time budgets as constants at the time-scale of the responses to manipulations, meaning that parents cannot increase their allocation to both provisioning and defense at the same time. This situation can arise from at least two different scenarios: (i) the time budgets might be genetically determined, similar to the parental effort variables in the model of Houston and Davies [9], or (ii) time budgets do change as in the model of McNamara et al. [10], but at slower time-scales than the responses to the manipulation. We do not model how the time budgets are determined, but in both of these scenarios, parents would experience the trade-off between parental effort and personal survival that is found in previous models, which would prevent the time budgets being arbitrarily large (see also Discussion).

The time allocated by the parents to offspring provisioning is denoted by 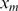 and 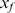, and the allocation to nest defense by 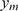 and 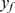 (the subscripts 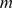 and 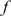 are for the male and the female, respectively), with 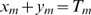 and 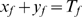. The production from the nest is the number of offspring surviving to the next breeding season. This varies as a function of the time allocated to defense and provisioning and is denoted by 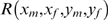. The nest production function will in general depend on a number of factors, including brood size, the developmental stage of the chicks, environmental variables, etc. Here, we are interested in short-term responses of parents to experimental manipulations, rather than the complete trajectories of their allocations, and therefore we assume that everything that affects the nest production function stays constant, except for the experimental manipulation. To reflect the dependence of nest production on both provisioning and nest defense, 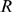 can be written as the product of two functions:
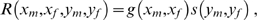
where 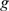 and 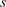 denote the growth rate and the survival rate of the brood as a result of parents' times spent provisioning and defending. Such a nest production function would arise when overwinter survival of juveniles is a function of the mass at fledgling (proportional to the growth rate 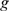), and the survival to fledgling is a function of nest predation. In this paper, we treat the nest production function 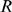 as exogenously specified and derive the responses of parents as a function of the function's shape. This approach can be complemented with a mechanistic one that derives the nest production function from the specific breeding ecology of a species and thus relates the responses of the parents to particular biological or environmental factors, such as temperature, food availability or predation pressure.

An individual's time allocation decision can only be based on the information it has. Therefore, the function 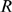 needs to be interpreted from the individual parent's perspective, as its *perception* of how the nest production depends on their allocations. Parents' perceptions of the nest production might differ from each other, and the real production function, depending on the information they possess about environmental factors (such as predation risk) and individual traits (such as effectiveness in foraging) that affect breeding success.

### The perfect family

For the perfect family case, we assume that parents communicate and arrive at a shared function 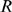. The optimal allocation problem is then to maximize a single function 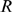 with respect to both parents' time allocations, subject to the budget constraints. There are two possible types of solutions: an interior solution meaning that both parents spend some time doing both tasks (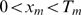 and 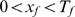), or a boundary solution where at least one of the parents devotes its time exclusively to a single task. Both parents spending their entire time budget doing different tasks corresponds to a complete division of labor. Evidence from birds with biparental care suggests that this is rare, so we focus mainly on interior solutions to the optimal allocation problem. The interior solutions can be found by taking the total derivative of 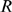 with respect to 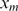 and 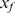 and setting it equal to zero:
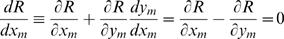
(1)

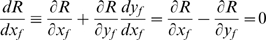
(2)


Here, we have incorporated the budget constraints into the first order conditions directly, by setting 
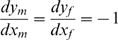
. We denote the optimal allocation by the parents that solve equations (1) and (2) by 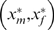, subject to second order conditions that ensure that the critical point is in fact a maximum, and that this maximum is a stable equilibrium of the behavioral dynamics following these objective functions [20]. For a discussion of behavioral stability under a different, discrete-time dynamics scenario, see [23].

### The almost perfect family

For the almost perfect family case, one needs to modify the first order conditions above. We now have two nest production functions, 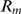 and 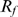, standing for the male's and female's perceptions of the nest production. This is because parents do not communicate with each other and therefore optimize with respect to their own perception of the nest production function. The first order conditions are then:
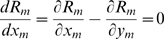
(3)

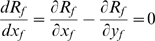
(4)


## Results

### Responses of parents to manipulations

In order for parents to change their allocations in response to an experimental manipulation, some aspect of the production function must change. For example, handicapping one of the parents might reduce how much that parent's time allocation to provisioning increases offspring survival, but leave other aspects of the production function unchanged. Alternatively, a begging call playback [12] might change the parents' perception of brood need, again amounting to altering (the perception of) how the time allocated to provisioning affects offspring growth. To express this mathematically, we introduce a parameter, 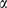, that modulates the shape of the nest production function. This parameter represents some property of the environment or of a parent that has an effect on how time allocations translate into offspring production. An experimental manipulation can then be represented as a change in 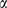. For example, handicapping the female can be represented by multiplying its foraging time 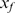, by a term 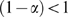 in the growth function 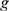, corresponding to a decrease in food brought to nest per unit time spent foraging. Increasing 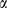 means more severe handicapping of the female. This example and an additional one is discussed further below.

### The perfect family

We want to calculate the change in parents' optimal allocations with 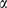, i.e. 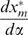 and 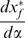. The ratio of these two derivatives, which we call the relative response ratio and denote by 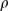, gives us how parents' optimal allocations change relative to each other. Thus, we have:
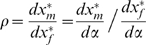
(5)


Almost all of the experimental studies measure only the food delivery to the nest, which corresponds to the allocation to provisioning in our model. Therefore, the quantity 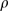 is what needs to be compared to experimental results.

Suppose the female is the manipulated parent. We can classify the different types of responses of the parents according to the sign and magnitude of the relative response ratio. Partial compensation occurs when the male and female adjust their allocations in opposite directions, but the male in a smaller magnitude than the female, i.e.:
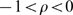
(6)


Similarly, overcompensation and matching are characterized by 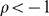 and 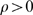, respectively.

To obtain the relative response ratio, we take the total derivative of equations (1) and (2) with respect to the experimental manipulation parameter 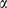, and solve the resulting two equations for 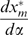 and 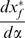. In this way, we obtain for the relative response ratio:
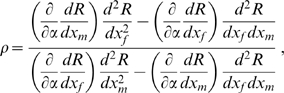
(7)


Note that this expression contains mixed derivatives of 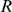 with respect to 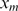 and 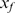, and 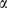 which means that the relative responses of the parents depend on how changing the value of 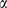 (in other words, the experimental manipulation) affects the sensitivity of the production function to the time allocations of the male and the female. Given a particular nest production function, the right-hand side of equation (7) evaluated at the optimum allocations, gives 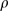 as a function of the experimental manipulation parameter 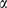. Note that 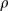 describes the relative changes in 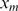 and 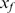. As such, we are not interested in the change in 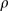 itself, rather, we want to know the value of 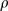 at a particular (non-manipulated) value of 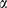. (Note that we assume an infinitesimal change in the parameter 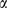; for real manipulations, the change in 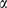 will be finite, so we would need to deal with differences rather than derivatives.)

We distinguish between two types of manipulations that are representative of the possible range. In the first one, the manipulation decreases the contribution of the female's provisioning effort, 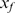, to the production function, i.e. 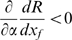, while increasing the contribution of the male's provisioning effort, 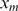, i.e. 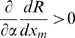. Such an effect would be produced by handicapping manipulations that decrease the female's ability to find food and with it the amount of food delivered per unit time spent foraging by the female, which increases the importance of the food that the male brings in. Thus, we call this type of experimental treatments *handicap manipulations*. The second type of manipulation is characterized by 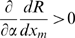 and 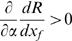, which would be the case when the real or perceived provisioning need of the brood is manipulated by, for example, food deprivation or playing begging calls to parents. We call such experimental treatments *need manipulations*. An example nest production function and two instances of manipulations that affect it in different ways are given below.

### Conditions for compensation in the perfect family

We focus on the conditions for a compensation response, i.e. the right-hand inequality in condition (6). This analysis is illustrative of the general pattern, namely that all three types of relative response ratios are possible, depending on the shape of the nest production function and the type of manipulation. Conditions for other types of responses can be found in a similar fashion.

The right-hand inequality in condition (6) is satisfied when the numerator and the denominator on the right hand-side of (7) have opposite signs. For handicap manipulations, a sufficient condition for a compensation response is that
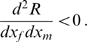
(8)


The mixed derivative of the production function with respect to its two inputs has an important meaning in economics. It measures whether the inputs are substitutes or complements for each other. If the mixed derivative is negative, that means that an increase in one of the inputs decreases the marginal value of the other input; such inputs are called substitutes. In our case, condition (8) says that the male and female both can substitute for changes in each other's allocation. It is easy to show that this will be the case when 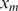 and 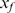, and 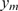 and 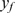 are substitutes for each other in the foraging and defense functions 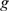 and 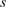, respectively. (In our notation, 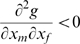 and 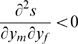.) In many bird species, we expect this to be true because both parents are usually capable of both provisioning and defense and thus one's effort can be substituted for the other's. On the other hand, a positive mixed derivative of 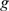 can come about when the overwinter survival is an accelerating function of the total food delivered. This can happen, for example, when there is some baseline level of food provisioning required for the basal metabolism of the nestlings, and anything above the baseline contributes to increased survival after fledging. This would make 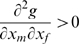, which enables a matching response. A similar argument can be made for the survival 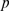 as a function of total time allocated to nest defense.

The situation changes under a need manipulation. Then, condition (8) no longer guarantees compensation, but it is a necessary condition for compensation. This means that even if the parents' allocations are substitutes for each other, the optimal response to a need manipulation can be in the same direction for both. Conversely, the inverse of inequality (8) guarantees a matching response. The implication of this change in the necessary and sufficient conditions mean that for some nest production functions, different types of manipulations can produce strikingly different types of relative responses.

### An example

We illustrate the differences in the relative response ratio predicted under two different types of manipulations with a specific nest production. We use the following functions for the provisioning and nest defense components, respectively:
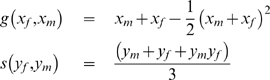



These functions are not meant to be realistic descriptions of the components of a nest production function. Rather, they are simple functions that incorporate the generally expected properties of a nest production function. The provisioning function 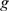 (similar form to the one used in [11]) increases with the sum of the parents' allocation, and displays diminishing returns from total time allocated to provisioning. The survival function 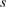 increases linearly with each parent's defense allocation and is scaled such that it is always between zero and one. The term 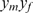 in the survival function both ensures that we have an internal solution to the optimization problem and also captures an aspect of defense behavior we do not explicitly model here. Generally, nestling survival will depend not only the amount of time spent on defense, but also the timing of the defense. For example, if both parents need to be present and defending the nest for effectively mobbing some nest predators. If parents do not coordinate their defense bouts, they would be together at the nest a fraction 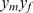 of the time, hence the product term.

To represent the two different types of manipulation, we parametrize the provisioning function in two different ways. For a handicap manipulation, we multiply 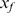 in 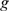 by a factor 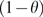, 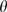 being the experimentally manipulated parameter. Increasing 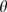 can be thought of as handicapping the female, so that it brings in less food per time spend foraging. This would decrease the contribution of female's provisioning allocation to the growth rate of the offspring. We assume for simplicity that the handicap on a female does not affect the contribution of her defense to the survival function 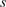. In reality, a handicap is likely to affect both the 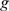 and 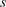 components of the nest production function. In such cases, experimental manipulation might not satisfy the part of our definition of the handicap manipulation that stipulates 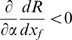. In general, one would need to verify that the effect of the manipulation on the provisioning function is sufficiently greater than the effect on the survival function, to satisfy this condition.

To model a need manipulation, we multiply the sum 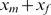 in 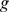 with a number 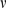 (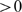). Increasing 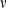 results in an increase in the slope of the provisioning function as a function of the total allocation to provisioning, as well as shifting its maximum towards higher provisioning efforts. Both of these effects correspond to an increase in the need (or potential to grow) of the brood [11], since a more hungry brood can ingest more food, and will benefit more from food received. The parameters 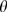 and 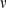 are two different instances of the generic experimental manipulation parameter 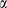 in equation (7).

**Figure 1:**
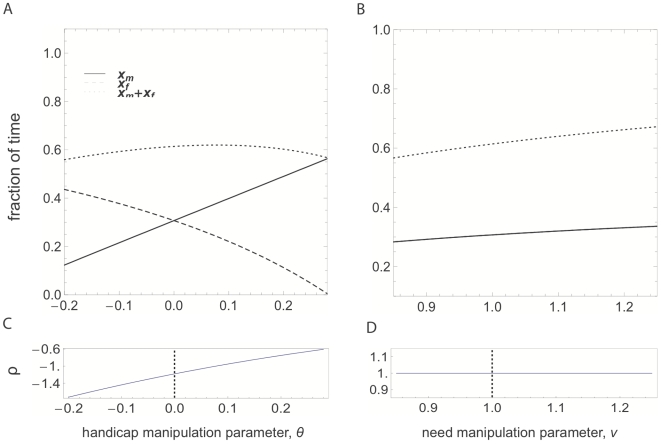
Response of the parents in the perfect family. Panels A and C are for a handicap manipulation; Panel A depicts parents' optimal allocations to foraging, 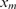 (solid line) and 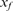 (dashed line), as well as total time spent foraging by the pair (dotted line) as a function of the handicap manipulation parameter, 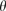. Panel C, on the other hand, plots the relative response ratio 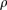, i.e. the ratio of the derivatives of 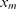 and 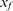 with respect to 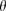. Note that in Panel A, 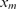 and 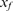 change in opposite directions; accordingly, 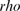 is negative over the range plotted here. Panels B and D depict the same for a need manipulation with the need manipulation parameter 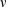. In Panel B, 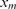 and 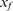 coincide; accordingly, the ratio of their derivatives is constant and equal to 1, corresponding to a matching response. The dotted vertical line in Panels C and D mark the non-manipulated nests for the two experiments; the sign of the relative response ratios 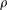 at these points differ between the two types of manipulations. The fact that 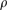 is constant and equal to 1 in Panel D is caused by the symmetry between the male and the female, and is not a generic feature of the perfect family model. If males and females are different in some respect, 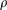 will be different than 1, and will vary.

Figure 1 shows how parents' allocations behave in the two manipulations. At 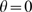 and 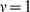, both parameterizations give production functions 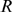 identical to each other. However, despite starting from the same point, handicapping the female (going right on Figure 1A) produces a partial compensation response, whereas increasing the need of the brood (going right on Figure 1B) results in a matching response. This is because in the handicap manipulation (Figure 1A), the overall need of the brood is not affected directly, while the female becomes less efficient in provisioning, so that the optimum allocation of the pair shifts towards the male shouldering more of the provisioning and the female more of the defense. In contrast, the need manipulation (Figure 1B) increases the overall need of the brood so that overall, provisioning becomes more important relative to defense, causing a shift in both parents' allocations towards more provisioning. Figures 1C and 1D further illustrate the responses by plotting the relative response ratio, 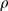. In the handicap manipulation (Figure 1C), 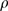 is negative and switches from overcompensation (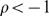) to partial compensation. In contrast, in the need manipulation, parents match each other's changes, resulting in a positive 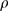. Thus, the perfect family behaves in radically different ways in response to different types of manipulations.

### The “almost perfect” family

The analysis in this case is similar to the perfect family case, but the fact that parents optimize individually with respect to their own perceptions of nest production changes the result markedly. Denoting the male's perception by 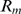 and the female's by 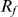, we again assume that the female is manipulated. As above, 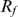 is a function of 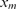, 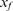 and the manipulated parameter, 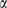, but now the male is not manipulated and parents do not exchange information. Therefore, the male does not see a change in 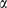, and 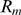 has no explicit 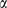-dependency. Taking the total derivative of the first order condition (3) for the male's allocation with respect to 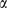, we get:
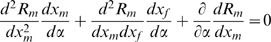
(9)


Now, the last term in this equation is zero, since 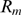 does not depend on 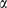 explicitly. Hence we can solve for the relative response ratio 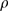 from this equation alone:
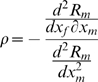
(10)


Thus, the relative response ratio in the almost perfect family only depends on 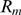, and not on how changes in 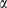 affect the female. (Note that we chose the female as the manipulated parent by convention only; if the male were to be manipulated, 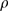 would depend only on 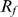.) The reason is simple: since the male does not see the manipulation, it cannot react to it directly. Therefore, the male reacts to the only change it can see, which is the change in female's allocation, 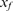. This is why we are able to compute the relative response ratio 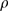 using only the male's first-order optimization equation, whereas we needed both first-order conditions in the perfect family case. Different manipulations will affect the absolute responses of the parents (i.e. 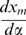 and 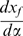), and we can compute both those using both first order conditions. However, the ratio of the absolute responses, 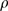, is constant, and independent from how the manipulation changes 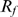.

**Figure 2:**
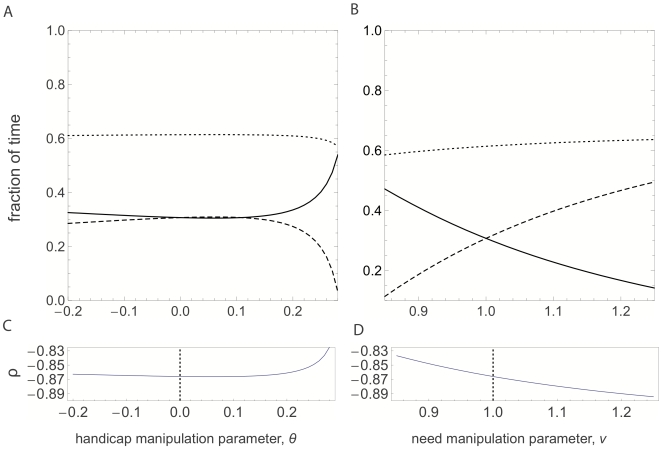
Response of the parents in the almost perfect family. Panels A and C are again for a handicap manipulation, and Panels B and D are for the need manipulation. Legend same as in Figure 1. The main difference here is that the almost perfect family condition predicts partial compensation in both cases. At the non-manipulated baseline marked with the dotted line, the relative response 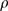 has the same value for both types of manipulations.

Looking at the condition for compensation (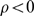), we find that the necessary and sufficient condition is now:
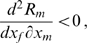
(11)since the second order condition for optimization ensures that the denominator in (10) is negative. Figure 2 depicts the response of an almost perfect family to the same manipulations described above. One can discern immediately that the almost perfect family respond differently than the perfect family. Whereas the perfect family exhibited either compensation or matching responses depending on the manipulation, the almost perfect family only exhibits a compensation response. For the handicap manipulation (Figure 2A), the female reduces its allocation to foraging, while the male compensates for this reduction. For the need manipulation (Figure 2B), the female increases its allocation to foraging, and the male compensates again, reducing its allocation to foraging. Figures 2C and 2D again depict the relative response ratio 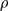: even though the shift of a given parent's allocation is in opposite directions for the two different manipulations, the relative response ratio is negative for both, and has the same value at the non-manipulated nest for the two manipulations. This feature of the almost perfect family is in stark contrast with the perfect family, where the relative response ratios under the different manipulations have opposite signs. This difference can be used to empirically distinguish between the perfect and almost perfect family cases (see Discussion).

## Discussion

Our aim in this paper is to present a simple model that can explain results from a number of manipulation experiments that fail to confirm the partial compensation response from earlier theory. Our explanation rests on the fact that empirical studies have mostly focused on only one component of parental care, provisioning of the offspring. However, breeding animals commonly face a trade-off between multiple tasks required for successful breeding [24]. Given such a trade-off, empirical studies that only focus on one component would not measure the total PE, and changes in the measured component due to manipulations do not necessarily imply changes in the total PE. To our knowledge, there is only one manipulation study that has measured time-budgets of parents and tracked how allocation to different tasks has changed. Markman et al. [17] found that handicapping female orange-tufted sunbirds (*Nectarinia osea*) reduced their nest-visiting rate, to which their partners responded by increasing theirs. While doing so, the males reduced the time they spend guarding the nest, indicating a trade-off between these two tasks. Future studies need to quantify time allocation of individuals to different parental tasks in order to gain a more complete picture of parents' responses to manipulations.

Another implication of our model is that the ecological determinants of nest production, reflected in the nest production function 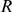, play a decisive role in determining how parents respond to manipulations. In particular, a nest production function where both parents can substitute for each other in both tasks tends to produce compensation type responses. On the other hand, when parents' allocations are complementary, matching responses become more likely. Thus, different species can exhibit different responses, depending on the details of their breeding ecology and the nest production function it results in. Similarly, how the manipulation actually affects the nest production also changes the results dramatically, possibly reversing the sign of the relative response ratio 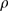 in the perfect family case (see below).

With this model, we also aimed to explore the process by which parents make their allocation decisions. Specifically, we modeled parents' responses as resulting from a behavioral objective function. We assumed that the behavioral objectives of the parents are entirely concordant, with both aiming to maximize nest production. This assumption can be viewed from three different perspectives. First, it can be seen as an empirical proposition: as detailed above, our model can be tested by quantifying the nest production function and comparing parents' responses to the prediction of our model. To model the responses of parents when they have a mix of common and private interests is beyond the scope of this paper, but would follow the same methodology as here. It is possible to build both perfect family (with communication) and almost perfect family (without communication) type models for the non-concordant objectives case as well. These models would yield expressions for the relative response ratio that depend on the private interests of the parents as well as their common interest in the nest production. Thus, testing the role of private and conflicting interests in the responses of parents to manipulations will likely require experimental methodologies that affect potential private interests separately from the nest production function.

The second perspective is to view our assumption as highlighting the theoretical possibility of parents having concordant objectives. Such a claim is at odds with the widely accepted notion that parents are in conflict with each other over parental decisions [25], and one might wonder how it can be justified. The key to answering this question is to note that in our model, behavioral objectives are proximate mechanisms for behavior, and are conjectured to evolve according to their fitness consequences, as proposed by Roughgarden et al. [19]. Even though behavioral objectives evolve to maximize fitness benefits to the individuals, they do not need to directly represent the fitness function. Whenever there is no lifetime genetic monogamy, evolutionary interests of parents will differ from each other, and may be in conflict. However, concordant objectives can result in positive behavioral feedbacks in parents' actions, which would lead both of them to invest more in acts that benefit each other, and both have higher fitness as a result. Recently, André and Day [21] analyzed the linear response rule model of McNamara et al. [10], and found that in finite populations, only efficient outcomes (i.e. ones upon which it is not possible to improve both individuals' payoffs simultaneously) can be evolutionarily stable. This finding is corroborated by Dekel et al. [22], who find that if the utility functions of individuals who play a non-cooperative game are allowed to evolve in a finite population, evolutionarily stable outcomes have to be efficient. Finally, Akçay et al. [20] show that efficient outcomes in a game with payoff conflict imply locally concordant objectives at the outcome. The combination of these results imply that evolution in finite populations should frequently lead to concordant objectives.

Finally, the third perspective is that conflict over total PE can also co-exist with concordant objectives at the nest. We do not model how parents determine their time budgets, which is the measure of total PE in our model. The fitness trade-off between PE and alternative reproductive opportunities that is the hallmark of sexual conflict models will be present at the determination of the time budgets, and can lead to conflict of interests. This conflict may well not be resolved. However, given a pair of time budgets, both individuals are interested in maximizing the returns from their time investment. Thus, even though parents might be in conflict over time budgets, they should have concordant interests while allocating their time budget for maximum nest production. We also note that we did not model potential conflicts between parents relating to hatching asynchrony [26] and differential investment in individual offspring [27]. An interesting question for future work is how the potentially different modes of decision making at the seasonal versus daily time-scales, as well as brood-level decisions versus decisions related to individual offspring interact with each other.

It is worth emphasizing again that these perspectives are not meant to imply that our model disproves the parents-in-conflict view. Our point is simply that there are strong theoretical and empirical reasons to devote close attention to the issue and the possibility of concordant objectives.

### The perfect vs. almost perfect family

Distinct from the question of the objectives driving parents' decisions is how parents with given objectives decide on their time allocations. In particular, we distinguish between the perfect and the almost perfect family models. In the almost perfect family model, parents do not communicate with each other except through changing their allocations. In contrast, the perfect family model allows for direct communication between parents about the manipulation itself. This leads to qualitatively different relative response regimes, since the optimal response to a manipulation of the brood's need is different than that to a handicap manipulation, and both parents can discern which is needed. Our model points out how these two cases can be distinguished from each other empirically.

In the perfect family, the relative response ratio of the parents can be positive, or negative, depending on the type of manipulation. In contrast, parents that do not communicate with each other when making allocation decisions will show the same relative response, regardless of the type of manipulation. An experiment can subject two groups of pairs from the same population to the two different types of manipulations described here and compare the relative response ratios between the groups. Previous studies suggest that parents of at least one bird species do show different responses to different types of manipulations: Sanz et al. [15] and Hinde [12] both manipulated pairs of great tits (*Parus major*), but in different ways. In response to their handicapping manipulation, Sanz et al. [15] documented a compensation response, while Hinde [12], using playbacks of begging calls, found that both parents increased their feeding effort. These results are consistent with the perfect family model and not with the almost perfect family, but they should be replicated in the same population to minimize possible confounding differences between populations.

It is also useful to compare our models to the work by McNamara et al. [10] and Johnstone and Hinde [11]. McNamara et al. stipulate that parents use genetically fixed, linear response rules which they predict to have negative slopes, corresponding to compensation responses. Johnstone and Hinde [11], in a model motivated in part by the matching response found by Hinde [12], show that the slope of the evolutionarily stable linear response rule can also be positive. The main ingredient in their model is uncertainty about the brood's real need, which generates evolutionarily stable response rules that under some parameter values prescribe matching behavior. Thus, the linear response rule approach also predict both matching and compensation by the parents, depending on the parameters describing parents' information about the brood's need. A prominent feature of these models is that the linear response rules are genetically fixed, meaning that a species that has evolved a positively sloped response rule will always respond to manipulations by a matching response (although the absolute direction might vary, e.g. parents might both decrease or increase their effort). This is very similar to what the almost perfect family predicts, and the underlying reason is the same in both cases: parents in both our almost perfect family and in the linear response rule model only react to changes in their partner's behavior. In other words, these models exclude communication between parents other than through changing efforts or allocations, whereas the perfect family model allows direct communication about the manipulation. Thus, the perfect family model also stands as an alternative to the response rule models [10], [11], and can be distinguished from them using the same empirical methodology described above.

To summarize, our goal in this paper was to present a model of behavioral decision making by parents that can explain the varied results from experimental studies that manipulate parents. Our model predicts the type of response as a function of the nest production function that encapsulates the information about the breeding ecology, and the type of manipulation carried out. We assume that parents have common behavioral objectives when making time allocation decisions in the nest, and show that given a trade-off between different parental tasks, varied responses of parents can be explained under this assumption. We suggest that future theoretical and empirical work should address the issue of whether parents have concordant or conflicting interests when making decisions at the nest. We provide two qualitatively different models for how the pair might act, and suggest a way in which these two models can be distinguished empirically. We hope that our model will renew interest on the diversity of ways in which parents make decisions and interact with each other.
